# Clinical Impact of a Common PDCD1 Germline Variant in DLBCL Patients Treated with CAR-T Cell Therapy

**DOI:** 10.3390/ijms27167356

**Published:** 2026-08-17

**Authors:** Katja Seipel, Marta Gonçalves Fonseca, Inna Shaforostova, Seok-Yun Lee, Ulrike Bacher, Thomas Pabst

**Affiliations:** 1 Department for Biomedical Research, University of Bern, 3008 Bern, Switzerland; 2 Department of Medical Oncology, Inselspital, Bern University Hospital, 3010 Bern, Switzerland; martha.gonsalvesfonseca@students.unibe.ch (M.G.F.); innaivanovna.shaforostova@insel.ch (I.S.); seok-yun.lee@insel.ch (S.-Y.L.); 3 Department of Hematology, Inselspital, Bern University Hospital, 3010 Bern, Switzerland; veraulrike.bacher@insel.ch

**Keywords:** diffuse large B-cell lymphoma (DLBCL), programmed cell death protein 1 (PD-1), B-lymphocyte antigen CD19, single nucleotide polymorphism (SNP), minor allele frequency (MAF), CAR-T cell therapy, FMC63-chimeric antigen receptor (FMC63-CAR), myeloid zinc-finger 1 (MZF1), activating enhancer binding protein 4 (AP4)

## Abstract

Chimeric antigen receptor CAR T-cells are effective immunotherapies for patients with relapsed or refractory diffuse large B-cell lymphoma (r/r DLBCL) with overall response rates of 63–84% and complete response rates of 43–54%. Programmed cell death protein 1 (PD-1) is a cell surface receptor with immune check-point function. There are several common germline variants of the *PDCD1* gene, including the linked single nucleotide polymorphisms (SNP) rs6710479 and rs2227981. Genetic variants of the immune-checkpoint regulator *PDCD1* gene may affect clinical responses to CAR-T cell therapy. In this retrospective single-center study, we assessed the prevalence and outcomes of the *PDCD1* gene variants rs6710479 and rs2227981 in r/r DLBCL patients undergoing CAR-T cell therapy. The linked SNP’s were prevalent in 68% of the studied DLBCL patients (CT-AG and TT-AA). In a retrospective comparative analysis, we observed differences in clinical outcomes in *PDCD1* CC-GG versus CT-AG and TT-AA carriers with one-year PFS rates of 80% versus 50% and 48% (*p* = 0.01), and two-year OS rates of 74% versus 55% and 33%, respectively (*p* = 0.001). In conclusion, common *PDCD1* germline variants may have influenced the treatment outcomes in FMC63-anti-CD19 CAR-T cell therapy, and the *PDCD1* major allele (CC-GG) may associate with a favorable response.

## 1. Introduction

Chimeric antigen receptor (CAR) T-cell therapies that target the antigen CD19 have been demonstrated to be efficacious in patients diagnosed with relapsed or refractory diffuse large B-cell lymphoma (r/r DLBCL). Patients receiving FMC-63 CAR-T products, including Axicabtagene Ciloleucel (Axi-cel) and Tisagenlecleucel (Tisa-cel), exhibited overall response rates ranging from 63 to 84%, with complete response rates ranging from 43 to 54%. These outcomes were found to be superior when compared to standard second or later line therapy, which consists of salvage chemotherapy followed by autologous stem cell transplantation (ASCT) or other salvage options [1,2,3,4,5,6]. Despite the notable complete response rates observed, a considerable proportion of patients treated with CAR-T therapy will eventually experience relapse or fail to attain complete remission [7,8]. The results of a multicenter clinical trial in which patients received Axi-cel revealed that 28% of patients with primary refractory disease and 30% of patients overall relapsed after a median follow-up period of 12.9 months [8].

While certain clinical characteristics may be associated with varying outcomes in response to CAR-T cell treatment [9,10,11], the mechanisms of resistance or relapse remain to be fully elucidated [12]. Genetic variants of the CD19 target antigen may contribute to explain differences in the response to CAR-T cell treatment [13]. Furthermore, genetic variants of immune-checkpoint regulators CTLA4 and LAG3 may influence T-cell activation and the response to CAR-T cell therapy [14,15]. Immune checkpoint receptors have been shown to modulate T-cell activity [16]. The presence of various *PDCD1* genetic variants has been demonstrated to be associated with an elevated risk of developing cancer, including PD-1.1 (rs36084323), PD-1.3 (rs11568821), PD-1.5 (rs2227981), PD-1.6 (rs10204525) and PD-1.9 (rs2227982) [17]. PD-1.5 (rs2227981) and PD-1.6 (rs10204525) have been associated with cancer risk and the treatment outcomes in AML with standard chemotherapy [18]. rs6710479 and rs2227981 are common *PDCD1* germline variants with European minor allele frequencies of 0.43 (ALFA), indicating a 32% European prevalence of CC-GG homozygotes, a 49% prevalence of CT-AG heterozygotes and a 19% prevalence of TT-AA homozygotes.

In this analysis, we investigated the prevalence of the common *PDCD1* germline variants rs6710479 and rs2227981 and the clinical outcomes in a retrospective study of a B-cell lymphoma cohort treated with FMC63 anti-CD19 CAR-T cell therapy.

## 2. Results

### 2.1. Characteristics of the Common PDCD1 Germline Variants

**Figure 1:**
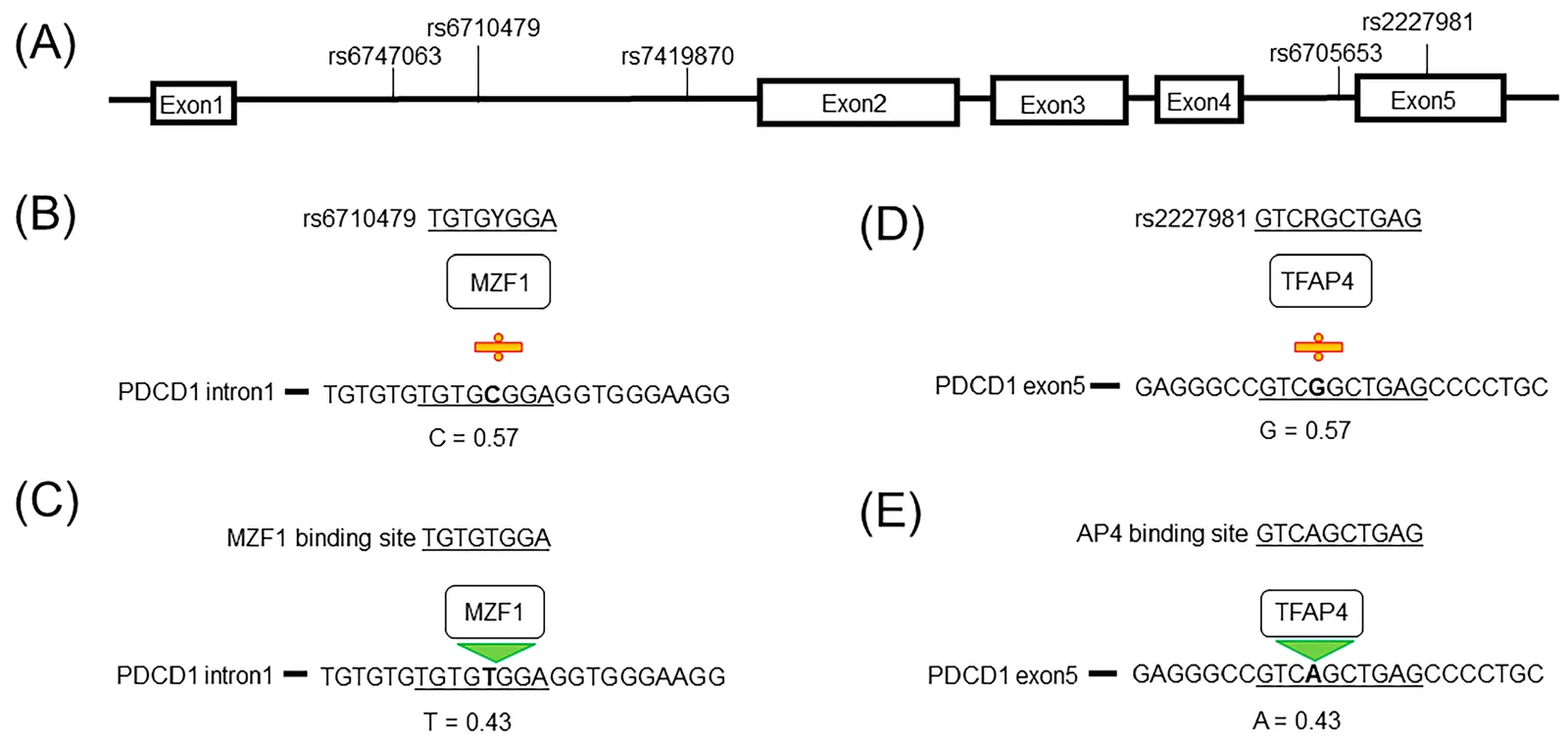
*PDCD1* gene and putative transcription factor binding sites MZF1 and TFAP4. (**A**) Schematic presentation of the *PDCD1* gene with common SNP sites, including rs6710479 and rs2227981. rs6710479 major allele (**B**), rs6710479 minor allele (**C**), rs2227981 major allele (**D**), and rs2227981 minor allele (**E**).

There are five common variants of the *PDCD1* gene with single nucleotide polymorphisms (SNP) rs6747063, rs6710479, rs7419870, rs6705653 and rs2227981 (four intronic and one exonic SNP (Table 1, Figure 1A)). The minor allele frequencies (MAF) in the European population are indicated on the allele frequency aggregator (ALFA) for rs6747063 (MAF 0.48), rs6710479 (MAF 0.43), rs7419870 (MAF 0.42), rs6705653 (MAF 0.42) and rs2227981 (MAF 0.43). With similar allele frequencies and close proximity within a 5400 bp genomic region, the five SNPs are inherited in one unit (linkage block). The linkage disequilibria (LD) of rs6710479 to rs6705653 and to rs2227981 were indicated at r^2^ = 0.86, D′ = 0.98, and r^2^ = 0.44, D′ = 0.93, respectively, on the Ensembl LD calculator [19], indicating a close genetic linkage with few recombination events. The genealogical age of the four intronic SNP variants was estimated at 18,000 to 50,000 generations (0.5–1.3 million years) on GEVA [20]. The exonic SNP rs2227981, due to higher selection pressure, may have evolved more recently, with an estimated genealogical age of 5000 to 15,000 generations (0.1–0.4 million years). The existence of two divergent *PDCD1* alleles may fit a model of human demographic history in which two ancestral populations diverged 1.5 million years ago and met in an admixture event 0.3 million years ago.

All of the five common *PDCD1* SNPs may host binding sites for transcription factors, pre-mRNA splice sites or splice factor binding sites, with differential affinity in the variant alleles. To identify pre-mRNA splice sites and splice factor binding sites, the SNP sequences were analyzed on ESE finder [21]. The splice sites and binding sites of splice factors SRSF1 (YGGAGGT in rs6710479, CRGCTGA in rs2227981, CRGCGTG in rs7419870), SRp40 (CCACTAY in rs6705653) and hnRNP A1 (TGYGGA in rs6710479) were conserved, indicating the parity of the associated splicing processes in both minor and major alleles of the *PDCD1* gene variants. To identify the transcription factor (TF) binding sites the SNP, core sequences were analyzed on TFBIND [22]. An MZF1 binding site (TGTGTGGA) was detected in the rs6710479 minor allele (score 0.86), indicating transcriptional regulation of the minor allele by MZF1 (Figure 1B,C). A TFAP4 binding site (GTCAGCTGAG) was detected in the rs2227981 minor allele (score 0.95), indicating transcriptional regulation of the minor allele by AP4 (Figure 1D,E). No differential TF binding sites were predicted in the rs6747063, rs7419870 and rs6705653 variants.

As *PDCD1* gene expression may be differentially regulated on the major and minor alleles, the following analysis was focused on rs6710479 and rs2227981. The sequences of the *PDCD1* gene intron1 and exon5 were determined in the peripheral blood of 112 DLBCL patients evaluated for CAR-T cell therapy at our center. Thirty five patients (32%) carried two major alleles (CC-GG), 51 patients (45%) had one minor allele each (CT-AG), and 21 patients (19%) carried two minor alleles (TT-AA). An additional 5 patients (4%) had a rare combination *PDCD1* genotype (3 CT-GG and 2 CT-AA). The *PDCD1* minor allele frequencies observed in the DLBCL cohort (Figure S1) were as expected in the European population at MAF 0.43 (ALFA sample size 22,082 and 60,844, respectively, https://www.ncbi.nlm.nih.gov/snp/, access date 6 August 2026) [23].

### 2.2. Baseline Clinical Characteristics of the DLBCL Patient Cohort

In this retrospective observational study, we analyzed a cohort of 112 patients with r/r DLBCL comparing three genetically variant groups with single nucleotide polymorphism in the *PDCD1* gene. The five patients with recombinant *PDCD1* genotypes were added to the heterozygous subgroup CT-AG. The median age at the time of initial diagnosis was 61 years, with 60 years in the CC-GG, 62 years in the CT-AG, and 63 years in the TT-AA subgroups (Table 2); 63% of the patients had de novo DLBCL, 37% transformed DLBCL, and 5% FL, with similar proportions in the three genetic subgroups. The majority of patients presented disease stage IV at the time of diagnosis according to the Ann-Arbor classification system. All patients received R-CHOP chemotherapy and up to four additional treatment lines prior to CAR-T cell therapy, and 38% of the patients received second line chemotherapy followed by ASCT prior to CAR-T as third line therapy. There was no difference in the proportion of patients with radiotherapy or ASCT in the three genetic subgroups.

### 2.3. Disease Features and CAR-T Cell Treatment

The median age at the time of CAR-T cell infusion was 67 years, with 64 years in the CC-GG, 68 years in the CT-AG, and 70 years in the TT-AA subgroups (Table 3). Half of the patients had progressive disease, confirmed by PET-CT; 39% of the patients received bridging chemotherapy; and 16% received bridging radiotherapy. Before CAR-T cell infusion, LDH levels and IPI were assessed. The median LDH levels were higher in the TT-AA subgroup (440 vs. 292 U/L, *p* = 0.14). High-risk IPI prevailed in the TT-AA subgroup (57% vs. 20%), while high-intermediate risk prevailed in the CC-GG subgroup (40% vs. 19%). All patients received lympho-depleting chemotherapy with fludarabine and cyclophosphamide before CAR-T cell infusion, and 55% of the patients were infused with Tisagenlecleucel (Kymriah©), 39% with Axicabtagene ciloleucel (Yescarta©) and 6% with Lisocabtagene Maraleucel (Breyanzi©, JCAR017).

### 2.4. Treatment Outcomes, Univariate and Multivariate Analysis

**Figure 2:**
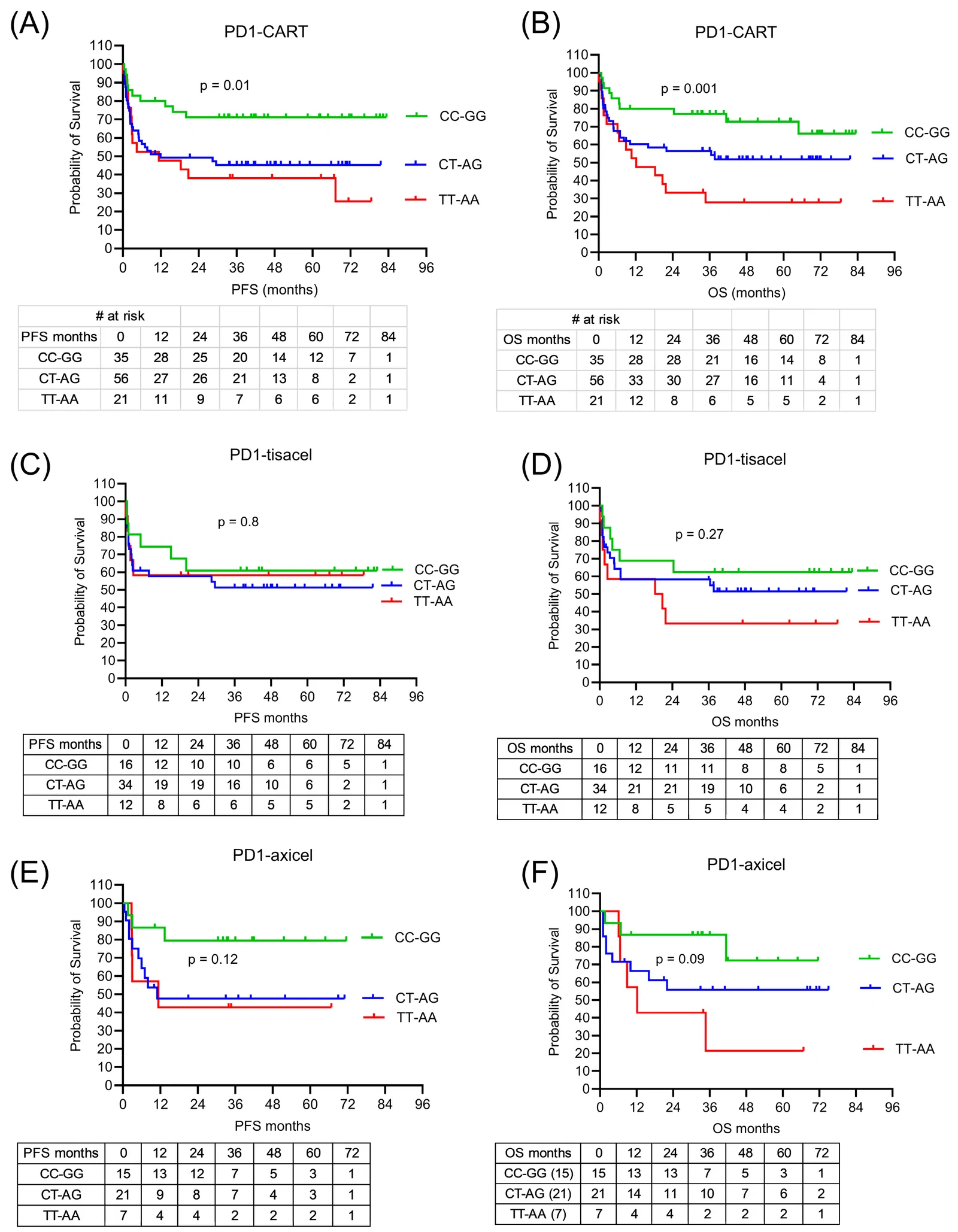
Clinical outcomes in DLBCL patients receiving FMC63 CAR-T cell therapy. Clinical outcomes in the entire cohort (**A**,**B**), in the subset treated with tisa-cel (**C**,**D**) and in the subset treated with axi-cel (**E**,**F**). PFS (**A**,**C**,**E**) and OS (**B**,**D**,**F**) according to *PDCD1* rs6710479-rs2227981 encoding *PDCD1* CC-GG, CT-AG or TT-AA. *p* values represent global three group log rank tests.

The outcomes of the CAR-T cell treatments were analyzed for the entire cohort and for the three genetic subgroups (Table 4). One to three days after CAR-T cell infusion, the majority of patients suffered cytokine release syndrome (CRS), at various grades, and five to seven days after CAR-T cell infusion a third of the patients suffered immune-effector associated neurotoxicity syndrome (ICANS). Nine to ten days after CAR-T cell infusion, peak plasma levels were detected, with a median 4531 copies per μg cfDNA. Six months after CAR-T cell infusion, CAR-T plasma levels were at median 100 copies per µg cfDNA. Response rates varied according to the *PDCD1* genotype, with 88%, 75%, and 71% objective response rates, and complete response rates of 74%, 46%, and 59% in the CC-GG, CT-AG and TT-AA genotype, respectively (Table 4). Relapse rates varied according to the *PDCD1* genotype, with 31%, 55% and 38% relapse in CC-GG, CT-AG, TT-AA, respectively. Death rates varied according to the *PDCD1* genotype, with 29%, 52% and 67% in CC-GG, CT-AG, TT-AA, respectively. Patients carrying the germline variant *PDCD1* CC-GG allele had better treatment outcomes, with median time PFS and OS over 6 years; the CT-AG group had median 1 year PFS and over 6 years OS, and the TT-AA group had median time PFS and OS of 1 year (Figure 2A,B). The one-year PFS rates differed significantly, with 80% in the CC-GG, 50% in the CT-AG, and 48% in the TT-AA subgroups (*p* = 0.01). The two-year OS rates differed significantly at 74% in the CC-GG, 55% in the CT-AG, and 33% in the TT-AA subgroups (*p* = 0.001). Similar effects were detected in the survival analysis according to the individual SNPs (Figure S2). To address the potential bias introduced by different CAR-T product types, the treatment outcomes were analyzed separately in tisa-cel- and axi-cel-treated patients. Patients carrying the germline variant *PDCD1* CC-GG allele had minimally better treatment outcomes in the tisa-cel treated subset (Figure 2C,D) and substantially better treatment outcomes in the axi-cel treated subset (Figure 2E,F). The multivariate analysis included parameters of possible impact on clinical outcomes, including age at the treatment start (>65 versus ≤65 years), disease status (transformed versus de novo DLBCL), type of CAR-T cell product (Axi-cel versus Tisa-cel), LDH levels, and IPI score at initial diagnosis. The multivariate analysis supported the trend observed in the univariate analysis for the *PDCD1* major alleles to be a prognostic indicator for treatment outcomes with HR = 0.34 at a *p*-value of 0.02 (Table 5).

## 3. Discussion

In this study, we analyzed the characteristics of five common SNP variants located in the *PDCD1* gene intron1 to exon5, in close genetic linkage. These variants may have offered the advantage of heritable diversity in immune responses across human populations, affecting susceptibility to infectious diseases and immune disorders. Due to the genetic linkage, the evaluated genetic combinations of rs6710479 and rs2227981 were largely restricted to two major alleles (CC-GG), one major and one minor allele (CT-AG), two minor alleles (TT-AA), and some rare combination genotypes (CT-GG and CT-AA).

In our DLBCL study cohort, the *PDCD1* gene variants rs6710479 and rs2227981 were prevalent at normal allele frequencies, indicating no association to cancer risk; however, high-risk IPI and elevated LDH levels prevailed in the TT-AA subgroup, indicating a tentative association of the *PDCD1* genotype with the lymphoma microenvironment. High-risk IPI was associated with depleted immune microenvironments, lower overall immune infiltration, and exhausted or immunosuppressive checkpoints [24]. DLBCL creates a hostile tumor microenvironment (TME) with multiple overlapping checkpoints [16]. The TME consists of T and B lymphocytes, tumor-associated macrophages (TAMs), myeloid-derived suppressor cells (MDSCs), cancer-associated fibroblasts (CAFs), and other components [25]. DLBCL cells promote T cell exhaustion through PD-1/PD-L1 interaction. PDCD1 expression on DLBCL cells is associated with poor prognosis [26]. PDCD1 is expressed on tumor-infiltrating lymphocytes and regulatory T-cells.

*PDCD1* gene variants rs6710479 and rs2227981 were associated with a clinical impact on treatment outcomes after CAR-T cell therapy. The one-year PFS rates differed, with 80% in the CC-GG, 50% in the CT-AG, and 48% in the TT-AA subgroups. The two-year OS rates differed, with 74% in the CC-GG, 55% in the CT-AG, and 33% in the TT-AA subgroups. The inferior outcomes in carriers of two minor alleles (TT-AA) were associated with the predicted transcription factor binding of MZF1 and TFAP4 to the minor allele. MZF1 and AP4 may upregulate the transcription of the *PDCD1* minor allele and induce higher levels of PDCD1 protein on the T-cell surface, leading to reduced T-cell activity and creating a tumor-supportive microenvironment. The proposed upregulation of *PDCD1* expression by MZF1 and TFAP4 is hypothetical and based on predicted binding sites without functional validation. Both MZF1 and TFAP4 expression were associated with an immunosuppressive microenvironment and resistance to anti-PDL1 immunotherapy [27,28,29].

**Figure 3:**
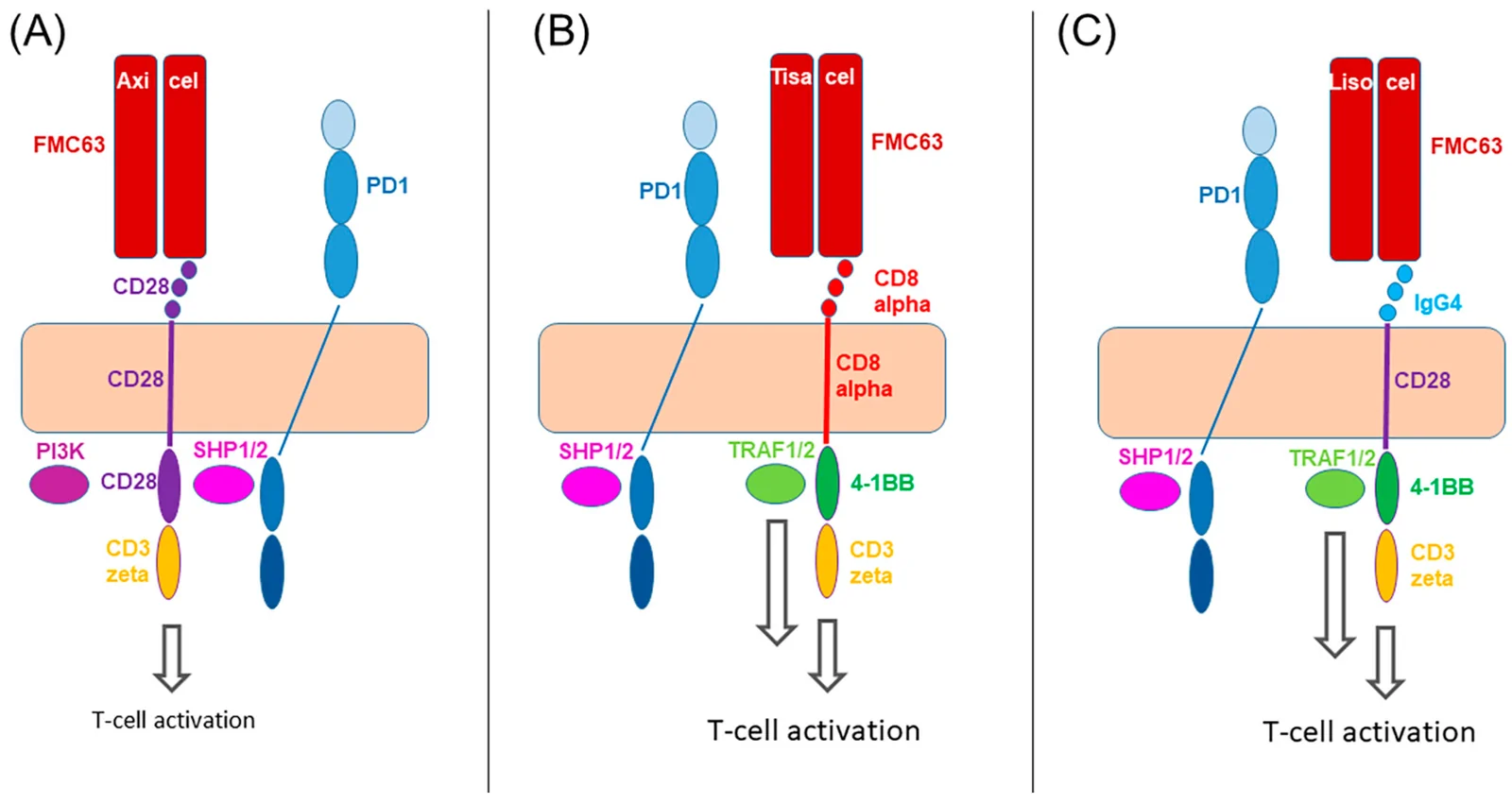
Schematic presentation of the three FMC63-based CAR-T cell products. The intracellular PDCD1 effector SHP1/2 (pink) may inhibit Axi-cel signaling by dephosphorylating the CD28 costimulatory domain (purple) (**A**). Tisa-cel (**B**) and Liso-cel (**C**) have a different costimulatory domain 4-1BB (green). All three CAR-T cell products have a CD3zeta T-cell activator domain (yellow).

To address the potential bias introduced by different CAR-T product types, the treatment outcomes were analyzed separately in tisa-cel and axi-cel treated patients. The association of the clinical impact of the *PDCD1* germline variants with treatment outcomes was substantial in axi-cel treated patients and minimal in tisa-cel treated patients, indicating a trend towards axi-cel specificity. Both CAR-T cell products feature an extracellular, CD19-targeting single-chain variable fragment FMC-63, with a CD28-originating hinge, a transmembrane and intracellular region in axi-cel, and a CD8-originating hinge and 4-1BB intracellular domain in tisa-cel (Figure 3). While CD28-based CAR-T cells exhibit strong initial potency and rapid expansion, 4-1BB-based CAR-T cells demonstrate greater persistence and long-term efficacy as well as distinct metabolic profiles [30,31]. While the T-cell costimulatory receptor CD28 is a primary target for PD-1 mediated inhibition [32], the T-cell co-stimulatory receptor 4-1BB acts via TRAF adaptor proteins to promote T-cell survival [33]. It is therefore feasible that PD-1 may have direct effects on the CD28-based axi-cel product but not on the 4-1BB based tisa-cel product. This working model (Figure 3) is hypothesis-generating and has not been validated.

The limitations of the study are the small cohort size and the variety of CAR-T cell products applied. Prospective validation in larger, ideally multi-center cohorts will be essential to confirm *PD-1-*dependent clinical impacts in DLBCL treated with CAR-T cell therapies and will allow for the analysis of interactions with other immune checkpoint regulators, including CTLA4 and LAG3, which both affect clinical outcomes in DLBCL with CAR-T cell therapy [14,15].

## 4. Materials and Methods

### 4.1. PDCD1 Gene Analysis

Genomic DNA was extracted from peripheral blood mononuclear cells (PBMCs) collected before CAR-T-cell infusion. DNA fragments were amplified using FIREPol (Solis Biodyne, Tartu, Estonia) and gene-specific primers covering *PDCD1* gene intron 1, forward primer 5′-CCACGGGCTGTGCTGGGATGA-3′, reverse primer 5′-CCGCCTTCTG AGGTCATGGGGT-3′, *PDCD1* gene exon 5, forward primer 5′-GGTCTGCAGAACACTGGTGGCCA-3′, and reverse primer 5′-CCCCCCGTGCCCTGTGT-3′. Sanger sequencing was performed at Microsynth, Switzerland. To identify splice factor binding sites, the SNP core sequences were analyzed on ESE finder [21]. To evaluate transcription factor binding sites, the SNPs core sequences were analyzed on TFBIND, Version 1 [22]. To evaluate the genetic associations of the SNPs, the linkage disequilibrium was analyzed on the Ensembl LD calculator [19]. To evaluate the genealogical variant age, the SNPs were analyzed on GEVA [20]. Minor allele frequencies were calculated on an Advanced Hardy-Weinberg Equilibrium Calculator [34].

### 4.2. Clinical Study

This retrospective single-center study included 112 lymphoma patients treated with CAR T cell therapy between January 2019 and January 2024 at the University Hospital of Berne, Switzerland. The following CAR-T cell products are included tisagenlecleucel (tisa-cel, Kymriah©, Novartis Pharmaceuticals, Basel, Switzerland), axicabtagene ciloleucel (axi-cel, Yescarta©, Kite-Pharma, Santa Monica, CA, USA), and lisocabtagene maraleucel (liso-cel, Brey-anzi©, JCAR017, Juno therapeutics, Seattle, WA, USA). The study was approved by the Ethics Committee Berne, Switzerland (decision number 2025-00853, date of approval 24 April 2025). The primary endpoints of the study were the clinical outcomes, which included the relapse rate, progression-free survival (PFS), and overall survival (OS). Secondary endpoints included the correlation of CAR T cell persistence and the administered CAR T cell product (tisa-cel vs. axi-cel), as well as the correlation with toxicities. CRS and ICANS clinical assessment and grading was performed following the American Society for Transplantation and Cellular Therapy (ASTCT) consensus grading.

### 4.3. Statistical Analysis

Progression-free survival (PFS) was defined as the time from CAR T-cell infusion to any of the following events: relapse, death or lost to follow-up. Overall survival (OS) was defined as the time from CAR T-cell infusion to the date of death. Progression-free survival (PFS) and overall survival (OS) were estimated using the Kaplan–Meier method and subsequently compared between groups using the log-rank test. The following variables have been included in the statistical analysis: age; sex; DLBCL transformation; the initial lymphoma stage; the number of previous therapy lines; previous stem cell transplantation; bridging therapy; the CAR T-cell product; the remission status at CAR T-cell infusion; the LDH level before CAR T-cell therapy; CRS; and ICANS. Categorical variables were analyzed using the fisher exact test or chi-square test. Non-parametric data were assessed using the Kruskal–Wallis test. Multivariable Cox proportional hazards regression models were performed to identify independent predictors for overall survival (OS) and progression-free survival (PFS). Variables were selected a priori based on clinical relevance. Missing data were handled using a complete-case analysis approach. The proportional hazards assumption was visually evaluated for all variables using log-log survival plots. Among the total cohort of 112 patients, 53 OS events (deaths) and 63 PFS events (progressions or deaths) were recorded and included in the respective multivariable analyses. All statistical analyses were conducted using GraphPad Prism (version 11.0.0; GraphPad Software, San Diego, CA, USA) and RStudio (version 2025.05.1 + 513; Posit PBC, Boston, MA, USA) for the purpose of conducting Cox regression analyses. A two-sided *p*-value of ≤0.05 was considered statistically significant.

## 5. Conclusions

In this retrospective observational study, we investigated the treatment outcomes related to the single nucleotide polymorphisms rs6710479 and rs2227981 in a DLBCL patient cohort receiving FMC63 anti-CD19 CAR-T therapy. Our data suggest that the germline *PDCD1* allele influences the treatment outcomes in FMC63-anti-CD19 CAR-T cell therapy and that the *PDCD1* major allele CC-GG predicts favorable treatment outcomes. To confirm the impact of the *PDCD1* germline allele in FMC63-CAR-T response, a larger retrospective study of FMC63 treated patients is required.

## Figures and Tables

**Table 1:** Characteristics of *PDCD1* gene common variants.

SNP	Genome	Location	nt	MAF	Variant Age (Generations, Years)
rs6747063	chr2:241,854,422	intron 1	C>G	0.48	21–39K, 0.6–1.1M
rs6710479	chr2:241,855,866	intron 1	C>T	0.43	18–26K, 0.5–0.7M
rs7419870	chr2:241,856,491	intron 1	A>G	0.42	23–50K, 0.6–1.3M
rs6705653	chr2:241,851,407	intron 4	C>T	0.42	22–40K, 0.6–1.1M
rs2227981	chr2:241,851,121	exon 5	G>A	0.43	5–15K, 0.1–0.4M

Minor allele frequency (MAF) in the current European population according to ALFA. K = thousand, M = million.

**Table 2:** Baseline clinical parameters, univariate analysis.

Parameter	All Patients	PDCD1 CC-GG	PDCD1 CT-AG *	PDCD1 TT-AA	*p*-Value
Patients, *n* (%)	112 (100)	35 (32)	56 (49)	21 (19)	
Male:Female (ratio)	64:48 (1.33)	19:16 (1.19)	32:24 (1.33)	13:8 (1.63)	0.85
Age at ID, years, median (range)	61 (24–79)	60 (28–78)	62 (24–79)	63 (43–75)	0.31
Initial diagnosis (ID)					0.90
DLBCL, *n* (%)	103 (92)	33 (94)	51 (91)	19 (90)	
de novo, *n* (%)	65 (63)	18 (55)	35 (69)	12 (63)	
transformed, *n* (%)	38 (37)	15 (45)	16 (31)	7 (37)	
FL, *n* (%)	6 (5)	1 (3)	3 (5)	2 (10)	
PMBCL, *n* (%)	2 (2)	1 (3)	1 (2)	0	
MCL, *n* (%)	1 (1)	0	1 (2)	0	
Initial disease stage (Ann-Arbor)					0.26
I, *n* (%)	4 (4)	1 (4)	2 (4)	1 (5)	
II, *n* (%)	17 (15)	4 (12)	8 (16)	5 (24)	
III, *n* (%)	17 (15)	8 (23)	6 (12)	3 (14)	
IV, *n* (%)	51 (46)	13 (37)	26 (53)	12 (57)	
na	23 (20)	9 (26)	14 (25)	0	
Chemotherapy lines					0.48
1	8 (7)	3 (9)	4 (7)	1 (5)	
2	39 (35)	8 (23)	22 (39)	9 (43)	
3–4	65 (59)	24 (69)	30 (54)	11 (52)	
Radiotherapy	47 (42)	14 (40)	24 (43)	9 (43)	0.97
ASCT	42 (38)	13 (37)	21 (38)	8 (38)	0.99

DLBCL: diffuse large B-cell lymphoma; FL: follicular lymphoma; PMBCL: primary mediastinal B-cell lymphoma; MCL: mantle cell lymphoma; na: not available; and ASCT: autologous stem cell transplant. * including CT-GG and CT-AA.

**Table 3:** Clinical characteristics and details of CAR-T cell treatments, univariate analysis.

Parameter	All Patients	PDCD1 CC-GG	PDCD1 CT-AG	PDCD1 TT-AA	*p*-Value
Patients, *n* (%)	112 (100)	35 (32)	56 (49)	21 (19)	
Age at CAR-T, years, median (range)	67 (25–82)	64 (29–80)	68 (25–82)	70 (51–78)	0.17
Time ID to CAR-T, years, median (range)	2.1 (0.4–26)	1.4 (0.5–26)	2.1 (0.5–26)	3.3 (0.8–20)	0.23
Remission status pre CAR-T					0.65
CR	8 (7)	1 (3)	6 (11)	1 (5)	
PR	36 (32)	14 (40)	14 (25)	8 (38)	
SD	4 (4)	2 (6)	2 (3)	0	
PD	53 (47)	15 (42)	28 (50)	10 (48)	
na	11 (10)	3 (9)	6 (11)	2 (9)	
Bridging chemotherapy	44 (39)	15 (43)	22 (39)	7 (33)	
Bridging radiotherapy	18 (16)	6 (17)	11 (20)	1 (5)	
LDH pre CAR-T (U/L), median	331	318	292	440	
(range)	(134–3949)	(145–1557)	(134–2355)	(204–3949)	0.82
Elevated LDH (>250 U/L), ***n*** (%)	64 (57)	20 (57)	29 (52)	15 (71)	0.34
Risk (IPI)					0.14
Low risk (0–1), *n* (%)	3 (3)	2 (6)	1 (2)	0	0.65
Low-intermediate risk (2), ***n*** (%)	8 (7)	2 (6)	5 (9)	1 (5)	0.45
high-intermediate risk (3), *n* (%)	37 (33)	14 (40)	19 (34)	4 (19)	0.27
High risk (4–5), *n* (%)	35 (31)	7 (20)	16 (28)	12 (57)	0.02
na, *n* (%)	29 (26)	10 (29)	15 (27)	3 (14)	0.50
CAR-T-cell product					0.24
Tisa-cel, Kymriah©	62 (55)	16 (46)	34 (61)	12 (57)	
**Axi-cel, Yescarta©**	43 (39)	15 (43)	21 (38)	7 (33)	
Liso-cel, Breyanzi©	7 (6)	4 (11)	1 (2)	2 (10)	

IPI: international prognostic index; CR: complete response; PR: partial response; SD: stable disease; PD: progressive disease; na: not available; and LDH: lactate dehydrogenase.

**Table 4:** Clinical outcomes after CAR T-cell therapy, univariate analysis.

Parameter	All Patients	PDCD1 CC-GG	PDCD1 CT-AG	PDCD1 TT-AA	*p*-Value
	112 (100)	35 (32)	56 (49)	21 (19)	
Cytokine release syndrome (CRS)	92 (82)	28 (80)	49 (88)	15 (71)	0.71
Grade 1	60 (54)	18 (51)	33 (59)	9 (43)	
Grade 2	27 (24)	8 (23)	14 (25)	5 (24)	
Grade 3	5 (4)	2 (6)	2 (4)	1 (5)	
Time to CRS, days, median (range)	2 (0–17)	2 (0–11)	3 (0–17)	1 (0–5)	0.14
ICANS	38 (34)	11 (31)	21 (38)	6 (28)	0.30
Grade 1	11 (10)	3 (9)	6 (11)	2 (10)	
Grade 2	7 (6)	3 (9)	3 (3)	1 (5)	
Grade 3	14 (13)	5 (14)	9 (16)	0	
Grade 4	6 (5)	0	3 (5)	3 (14)	
Time to ICANS, days, median (range)	6 (0–39)	7 (0–39)	5 (1–15)	5 (1–10)	0.14
CAR-T plasma peak, median copies per µg cfDNA (range)	4531 (30–218,384)	4968 (239–218,384)	4356 (37–64,424)	6381 (30–127,942)	0.46
CAR-T persistence 6 mo, median (range)	100 (0–4061)	97 (0–4061)	96 (0–2191)	129 (0–2317)	0.97
CRP peak, mg/L median (range)	43 (3–328)	35 (3–323)	48 (3–285)	70 (3–328)	0.64
Time to CRP peak, days, median (range)	3 (0–28)	3 (0–15)	3 (0–28)	2 (0–15)	0.22
IL-6 peak, pg/mL, median (range)	606 (4–157,198)	559 (4–50,055)	653 (9–157,198)	593 (7–142,173)	0.94
Time to IL-6 peak, days, median (range)	4 (0–39)	5 (0–39)	4 (0–26)	4 (1–39)	0.74
Ferritin peak, ug/L, median (range)	1211 (99–13,393)	1201 (161–9791)	1358 (271–13,393)	919 (99–12,398)	0.56
Time to Ferritin peak days, median (range)	10 (0–44)	10 (0–45)	10 (0–44)	10 (0–21)	0.97
Therapy response					0.11
ORR, *n* (%)	88 (79)	31 (88)	42 (75)	15 (71)	0.19
CR, *n* (%)	61 (55)	26 (74)	25 (46)	10 (59)	0.02
PR, *n* (%)	27 (24)	5 (14)	17 (31)	5 (29)	0.24
SD, *n* (%)	5 (4)	1 (3)	3 (6)	1 (6)	
PD, *n* (%)	14 (13)	3 (9)	9 (17)	2 (10)	0.65
na, *n* (%)	5 (4)	0	2 (4)	3 (14)	
Relapse, *n* (%)	50 (45)	11 (31)	31 (55)	8 (38)	0.06
Death, *n* (%)	53 (47)	10 (29)	29 (52)	14 (67)	0.01
one year PFS rate, n (%)	66 (59)	28 (80)	28 (50)	10 (48)	0.01
two year OS rate, n (%)	64 (57)	26 (74)	32 (55)	6 (33)	0.001

ICANS: Immune Effector Cell-Associated Neurotoxicity Syndrome; cfDNA: cell-free DNA; CRP: C-reactive protein; ORR: objective response rate; CR: complete response; PR: partial response; SD: stable disease; PD: progressive disease; na: not available; PFS: progression-free survival; and OS: overall survival.

**Table 5:** Clinical outcomes after CAR-T cell therapy, multivariate analysis.

	PFS		OS	
Predictors	HR (95% CI)	*p*-Value	HR (95% CI)	*p*-Value
***PDCD1* CC-GG vs. TT-AA**	0.61 (0.2–1.7)	0.35	0.34 (0.1–0.8)	0.02
***PDCD1*** CT-AG vs. TT-AA	1.22 (0.5–3.0)	0.66	0.74 (0.4–1.5)	0.42
Age > 65 vs. ≤65	0.81 (0.4–1.6)	0.52	1.35 (0.8–2.4)	0.30
Transformed vs. de novo	1.15 (0.6–2.2)	0.67	0.54 (0.3–1.0)	0.06
Axi-cel vs. Tisa-cel	1.12 (0.6–2.1)	0.72	0.84 (0.5–1.5)	0.56
LDH > 250 vs. <250 U/L	1.36 (0.7–2.6)	0.35	1.56 (0.8–3.0)	0.18
IPI ≤ 1 vs. ≥2	0.64 (0.3–1.2)	0.18	0.8 (0.4–1.5)	0.48

## Data Availability

Data available on request due to restrictions, privacy and ethics.

## Supplementary Materials

The following supporting information can be downloaded at https://www.mdpi.com/article/10.3390/ijms27167356/s1.
