# Supplementary material for: Clinical Impact of a Common PDCD1 Germline Variant in DLBCL Patients Treated with CAR-T Cell Therapy
Source: Int J Mol Sci. 2026 Aug 17;27(16):7356. doi: 10.3390/ijms27167356 (PMC13513131; doi:10.3390/ijms27167356)
Supplement: Supplementary file 1 [file ijms-27-07356-s001.zip › ijms-4457922-supplementary.pdf]

Supplementary Materials

Clinical Impact of a common PDCD1 germline variant in DLBCL patients treated with CAR-T Cell Therapy

Katja Seipel[1,2*], Marta Gonçalves Fonseca[2], Inna Shaforostova[2], Seok-Yun Lee[2], Ulrike Bacher[3], Thomas Pabst[2*]

(A)

DLBCL Group Results (N = 112)

rs2227981 Allele Frequencies: p = 0.567, q = 0.433

Genotype	Observed	Expected	%
GG	38	36	33.93%
AG	51	55	45.54%
AA	23	21	20.54%

HWE Test: χ^2 = 0.5912, p = 0.4420 (IN HWE)

(B)

DLBCL Group Results (N = 112)

rs6710479 Allele Frequencies: p = 0.563, q = 0.438

Genotype	Observed	Expected	%
CC	35	35.44	31.25%
CT	56	55.13	50.00%
TT	21	21.44	18.75%

HWE Test: χ^2 = 0.0282, p = 0.8666 (IN HWE)

(C)

SNP	Project	Population	sample size	ref allele	alt allele
rs6710479	ALFA	European	22082	T=0.43406	C=0.56594
rs6710479	gnomAD	European	78496	T=0.42433	C=0.57567
rs2227981	ALFA	European	60844	A=0.43304	G=0.56696

Figure S1. Minor allele frequencies of *PDCD1* rs222781 and rs6710479 in the B-cell lymphoma cohort. Advanced Hardy-Weinberg Equilibrium Calculator (A) rs227981 (B) rs6710479 and NCBI reference report accessed on August 8, 2026 (C).

(A)

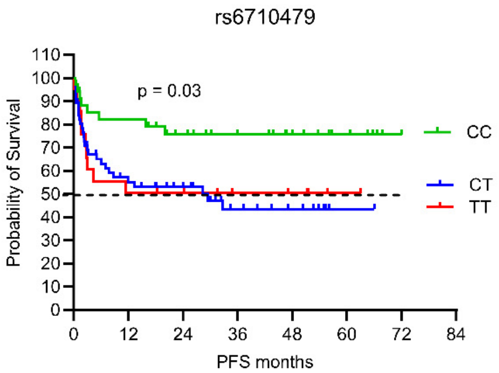

PFS months	0	12	24	36	48	60	72
CC	35	28	22	17	12	8	1
CT	56	28	21	12	8	2	1
TT	21	11	8	6	5	1	1

(B)

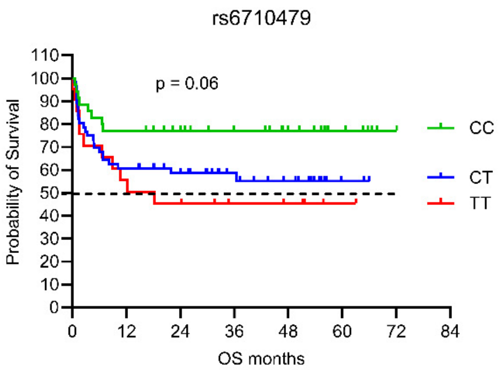

OS months	0	12	24	36	48	60	72
CC	35	28	23	19	14	8	1
CT	56	35	26	18	13	3	1
TT	21	12	10	6	5	2	1

(C)

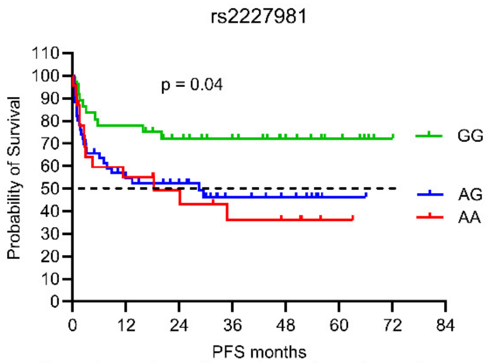

PFS months	0	12	24	36	48	60	72
GG	38	29	23	18	12	8	1
AG	51	26	20	11	8	2	1
AA	23	13	10	6	5	2	1

(D)

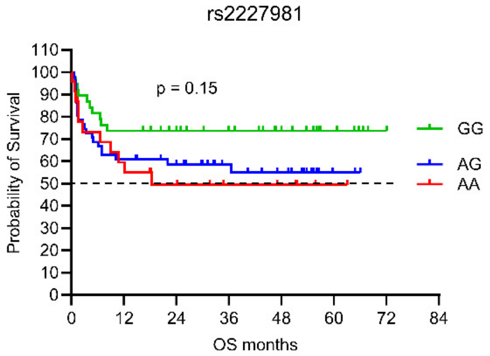

OS months	0	12	24	36	48	60	72
GG	38	29	24	20	14	8	1
AG	51	32	25	17	13	3	1
AA	23	14	10	6	5	2	1

Figure S2. Clinical outcomes in DLBCL patients receiving FMC63 CAR-T cell therapy. PFS (A,C) and OS (B,D) according to *PDCD1* rs670479 (A,B). PFS and OS according to *PDCD1* rs2227981 (C,D). p values represent global three group log rank tests.
